# Similar Profile and Magnitude of Cognitive Impairments in Focal and Generalized Epilepsy: A Pilot Study

**DOI:** 10.3389/fneur.2021.746381

**Published:** 2022-01-12

**Authors:** Helena Gauffin, Anne-Marie Landtblom, Patrick Vigren, Andreas Frick, Maria Engström, Anita McAllister, Thomas Karlsson

**Affiliations:** ^1^Department of Neurology, Faculty of Medicine and Health Sciences Linköping University, Linköping, Sweden; ^2^Department of Biomedical and Clinical Sciences, Faculty of Medicine and Health Sciences Linköping University, Linköping, Sweden; ^3^Department of Neuroscience, Neurology, Uppsala University, Uppsala, Sweden; ^4^Neurology Division, Clinic of Medical Specialist, Motala General Hospital, Motala, Sweden; ^5^Center for Medical Image Science and Visualization, Linköping University, Linköping, Sweden; ^6^The Beijer Laboratory, Department of Neuroscience, Psychiatry, Uppsala University, Uppsala, Sweden; ^7^Department of Medical, Health and Caring Sciences, Linköping University, Linköping, Sweden; ^8^Division of Speech Language Pathology, Department of Clinical Science, Intervention and Technology, Karolinska Institute, Stockholm, Sweden; ^9^Women's Health and Allied Health Professionals Theme, Medical Unit Speech and Language Pathology, Karolinska University Hospital, Stockholm, Sweden

**Keywords:** epilepsy, cognition, language, focal epilepsy, generalized epilepsy, quality of life, self-esteem

## Abstract

**Introduction:** Cognitive impairments in epilepsy are not well-understood. In addition, long-term emotional, interpersonal, and social consequences of the underlying disturbances are important to evaluate.

**Purpose:** To compare cognitive function including language in young adults with focal or generalized epilepsy. In addition, quality of life and self-esteem were investigated.

**Patients and Methods:** Young adults with no primary intellectual disability, 17 with focal epilepsy and 11 with generalized epilepsy participated and were compared to 28 healthy controls. Groups were matched on age (mean = 26 years), sex, and education. Participants were administered a battery of neuropsychological tasks and carried out self-ratings of quality of life, self-esteem, and psychological problems.

**Results:** Similar impairments regarding cognitive function were noted in focal and generalized epilepsy. The cognitive domains tested were episodic long-term memory, executive functions, attention, working memory, visuospatial functions, and language. Both epilepsy groups had lower results compared to controls (effect sizes 0.24–1.07). The total number of convulsive seizures was predictive of episodic long-term memory function. Participants with focal epilepsy reported lower quality of life than participants with generalized epilepsy. Lowered self-esteem values were seen in both epilepsy groups and particularly in those with focal epilepsy. Along with measures of cognitive speed and depression, the total number of seizures explained more than 50% of variation in quality of life.

**Conclusion:** Interestingly, similarities rather than differences characterized the widespread cognitive deficits that were seen in focal and generalized epilepsy, ranging from mild to moderate. These similarities were modified by quality of life and self-esteem. This study confirms the notion that epilepsy is a network disorder.

## Introduction

Cognitive function is often impaired in epilepsy, but the degree of impairment can range from subtle symptoms to significant disability. The underlying cognitive networks can be affected for different reasons. An underlying disease, genetic predisposition, structural changes, anti-seizure medication (ASM), metabolic alterations, duration of illness as well as the seizure-frequency are all factors that may contribute to cognitive impairment (1, 2). However, potential differences in cognitive impairment between different classifications of epilepsy i.e., focal and generalized epilepsy, are not well-investigated. In addition, long-term emotional, interpersonal, and social consequences of these underlying disturbances add to the cognitive challenges facing the individual (3, 4). Impairments of self-esteem, Quality of Life (QoL), communication and social life in young adulthood are described, results which are also being supported by previous studies from our group (5–9).

Studies have demonstrated cognitive impairments in both focal and generalized epilepsy, but only few studies have compared cognition in such epilepsy groups (10–14). Cognition has been most thoroughly studied in therapy resistant *focal epilepsies*, in particular temporal lobe epilepsy (15). Individuals with focal epilepsy have shown impairments in different cognitive domains, such as long-term episodic and semantic memory, language, and executive functions (16, 17). The interictal impairment of function in focal epilepsy may correspond to the localization of the corresponding underlying focal lesion (18). Temporal lobe epilepsy is associated with memory deficits (19), frontal lobe epilepsy with executive deficits (difficulties in response planning, selection, execution, inhibition, and evaluation) and attention problems (20) and parietal and occipital lobe epilepsies with reduction in global cognitive capacity, memory, and executive function (15). However, patients with focal epilepsy may also evidence impairments in cognitive domains typically related to other cortical regions than the actual site of the ictal focus (21–23).

Cognitive functions in *generalized epilepsy* have mostly been studied in small specified groups (24). Language abnormalities have been found among children with generalized as well as focal epilepsy (25). In adults with genetic generalized epilepsy impaired results for cognitive ability, acquired knowledge, long-term retrieval and information processing were demonstrated in one study (26) and attention was impaired in another (27). Executive functions were also described to be negatively affected in juvenile myoclonic epilepsy (28), but also in other generalized epilepsies (29, 30).

A recent interesting publication indicates that the lesion based model for explaining impairment of cognition in focal and generalized epilepsy is imprecise and that the clinical presentations are more heterogeneous (31). Still, knowledge remains limited regarding similarities and differences between focal and generalized epilepsy, especially in adults.

Cognitive function, psychological well-being, and QoL have a complex interaction. The impact of causal and modifying factors for QoL is far from being fully understood (4, 32). For example, for some patients the cognitive side-effects of ASMs can be more debilitating than the seizures and therefore contribute to low QoL (33). The most evident determinant for QoL in epilepsy is recent epileptic seizures (4, 34). However, long-time seizure-free patients also describe impaired QoL (35). Memory impairment, even subjective memory problems, has been shown to have an impact on QoL in epilepsy (36–38). Depression, impairment of executive functioning and language have also all been recognized as risk factors for affecting QoL negatively (39, 40). However, more research is needed to understand how cognition in epilepsy and QoL interact.

We planned to compare cognitive functions in patients with generalized and focal epilepsy respectively, and expected some differences between the two groups. However, when crudely analyzing the preliminary data, another picture emerged, i.e., with many similarities instead of differences. In a situation as the present, a persistent problem with statistical procedures involving testing and possibly rejecting of the null hypothesis (i.e., the absence of a difference) is that these methods are unable to quantify the extent to which null results actually support the null hypothesis (41). This becomes problematic when at least some comparisons between epilepsy patients with different subtypes are expected to fail to reach conventional criteria for statistical significance (i.e., a *p*-value smaller than 0.05). This outcome does not tell us that there is no difference but that the specific model tested is not compatible with the data. One way to mitigate this problem and to estimate the evidence in favor of the absence of differences is to employ Bayesian methods, which was applied in this work (42). A Bayesian analysis can quantify the evidence for any hypothesis, including the null hypothesis.

### Aims

This study intended to elucidate the patterns of similarities and differences of cognitive functions between young adults with focal and generalized epilepsy without gross primary intellectual disability. We also wanted to elucidate the cognitive mechanisms in these epilepsy subgroups. Furthermore, QoL and self-esteem were investigated to explore if there is a specific relationship between cognition and QoL/self-esteem.

## Methods

### Participants

Twenty-eight young adults with focal or generalized epilepsy at two centers in Sweden (University Hospital in Linköping and Motala General Hospital) were included in the study. The diagnosis was determined by semiology, MRI, EEG and, when applicable, ictal EEG registration. Inclusion criteria were 18–35 years of age; Swedish as first language without reported language dysfunction; minimum 9 years of elementary schooling without special educational needs; epilepsy diagnosis of focal or generalized epilepsy. Exclusion criteria: vagal nerve stimulator or other electrical implant that could interfere with MRI investigation; the use of psychoactive drugs other than ASM; unclassified or cryptogenic epilepsy; and/or history of psychogenic non-epileptic seizures. Twenty-eighth healthy controls (matched to epilepsy participants with respect to age, sex, educational level, and handedness) were recruited through advertisement. Demographics are given in Table 1.

**Table 1:** Clinical information regarding participants with epilepsy in a study on cognition in Sweden (*n* = 28).

**Sex**	**Age (years)**	**Education (years)**	**Onset (years)**	**Classification (simplified)**	**Etiology**	**Seizure-frequency**	**ASM (*n*)**	**Brain surgery**
F	20	12	6	Generalized epilepsy tonic-clonic seizures only	Unknown	Generalized annually	2	No
F	21	11	16	Focal seizures	Unknown, but suspect structural lesion frontotemporal right	Generalized annually, Focal weekly	3	No
M	32	14	8	Focal seizures	Mesial sclerosis temporal left	No seizures	2	Anterior temporal lobe resection left
F	27	14	17	Generalized epilepsy tonic-clonic seizures only	Unknown	Generalized weekly	2	No
F	29	14	6	Focal seizures	Mesial sclerosis temporal right	Focal weekly	3	Anterior temporal lobe resection right
M	20	12	16	Focal seizures	Cavernous hemangioma temporal left	Generalized annually, Focal monthly	3	No
M	22	12	15	Generalized tonic-clonic seizures only	Unknown	Generalized annually	2	No
F	32	15	27	Focal seizures	Status post-surgery oligodendroglioma temporal left	Focal weekly, Generalized 2 per month	3	Temporal lobe resection left
M	33	12	26	Focal seizures	Status post arteriovenous malformation tempero-occipital right	Several focal annually. Generalized annually	2	Resection tempero-occipital right
F	25	16	17	Focal seizures	Unknown, suspect structural lesion temporal left	No seizures	2	No
F	26	13	7	Focal seizures	Mesial sclerosis temporal right	Focal weekly	2	No
F	35	12	15	Juvenile myoclonic epilepsy	Genetic	Generalized monthly. Some myoclonic and absence seizures	3	No
F	22	12	0	Generalized epilepsy with febrile seizures plus	Genetic	No seizures	2	No
M	32	15	18	Generalized epilepsy, tonic-clonic seizures only	Unknown	No seizures	2	No
F	18	12	8	Focal seizures	Unknown, suspect structural lesion temporal left	Focal annually, Generalized annually	2	No
M	21	12	6	Focal seizures	Status post encephalitis, suspect lesion temporal left	Focal monthly, Generalized annually	2	No
F	19	10	1	Focal seizures	Suspect migration abnormality central-parietal right	Focal annually	2	No
F	22	13	17	Juvenile absence epilepsy	Unknown	Generalized annually	2	No
M	32	14	22	Generalized epilepsy tonic-clonic seizures only	Unknown	Generalized annually	2	No
M	29	10	4	Focal seizures	Status post-surgery cyst parietal right	Generalized annually	3	Resection parietal right
M	23	14	8	Focal seizures	Schizencephaly tempero-parietal right	No seizure	2	No
M	27	12	19	Generalized epilepsy tonic-clonic seizures only	Unknown	No seizure	1	No
F	23	13	17	Juvenile myoclonic epilepsy	Genetic	No seizure	1	No
F	24	14	22	Focal seizures	Unknown, suspect structural lesion frontal right	Focal monthly, Generalized annually	1	No
F	29	16	10	Focal seizures	Mesial sclerosis temporal left	Focal monthly, Generalized annually	2	Temporal lobe resection left
M	20	13	19	Focal seizures	Oligodendroglioma temporal left	Focal weekly, Generalized annually	1	No
F	27	19	14	Focal seizures	Unknown, suspect structural lesion temporal right	Focal monthly	1	No
M	29	16	9	Generalized epilepsy, tonic-clonic seizure only	Unknown	No seizure	2	No

*F, female; M, male; ASM, Anti-Seizure Medication; Seizure Frequency is approximate*.

### Materials and Procedures

#### Episodic Long-Term Memory

Episodic long-term memory (LTM) was evaluated by means of the *Rey Auditory Verbal Learning Test* (RAVLT) (43). We also used *the Rey Complex Figure Test* including copy, delayed recall and recognition tests (38).

#### Executive Functions

*Trail Making Test* (TMT) test A and B were used (43). The TMT requires a participant to connect an ordered set of 25 items spread out on the test sheet as quickly as possible without making errors.

#### Attention

Three tasks to address attention were used, including short-term memory capacity: *Digit Span, The Auditory Consonant Trigrams*, and *the Listening Span task*. Three tasks that would reflect different aspects of attention and working memory were used: digit span, reading span, and the Auditory Consonant Trigrams task, a psychometric implementation of the Brown-Peterson paradigm. Although all three tasks reflect attention and working memory, digit span reflects more automatized skills, whilst reading span involves more of executive attention skills, and emphasizes storage in the working memory (44).

*Digit span*, including forwards and backwards recall, was taken from the fourth edition of the Wechsler Adult Intelligence Scale, WAIS-IV (45).

The *Auditory Consonant Trigrams* test (46) taxes forgetting in primary or short-term memory. Participants are asked to repeat three consonants (e.g., “CJD”) following distractor-filled time intervals of 9, 18, or 36 s.

*Listening span* (47) reflects the number of words the participant can remember when being asked to recall the last word of each sentence in a passage that they heard.

#### Visuospatial Functions

Two tasks were used to assess visuospatial functions, the copy task included in *Rey Complex Figure Test* and *the Block Design task*.

In the *Copy tas*k (43) a complex figure is reproduced on a blank paper, without timing; the task is not timed. The *Block Design* task is part of the WAIS-IV (45) and is a valid indicator of spatial ability.

#### Language

Language capacity was evaluated by means of two tasks: the *Controlled Oral Word Association Test* and the *BeSS* (“BEdömning av Subtila Språkstörningar”; “Assessment of subtle language impairments”).

The *Controlled Oral Word Association Test* (COWAT) is a verbal fluency test (48). In the Swedish version, the participant is provided phonemic cues, the letters F, A, and S, and asked to generate as many different words (excluding proper names), as possible, within 1 min.

The *BeSS* (49) examination was developed to provide an in-depth assessment of patients with milder language deficits that may go unnoticed with the use of traditional procedures used to detect dysphasia. Also mild but significant language problems may not be apparent to the patient or the medical examiner. The *BeSS* battery investigates the following abilities: 1. Repetition of long sentences, 2. Sentence construction, 3. Inference (text understanding), 4. Comprehension of complex grammatical constructions, 5. Comprehension of ambiguous sentences, 6. Comprehension of metaphors, 7. Definition of words. Each subtest comprising 10 questions can result in a maximum of 30 points which give a total of 210 points. Inter-rater reliability varies between 0.93 and 0.99 for the different subscales (50). Intra-rater reliability is 0.96 for the total score and ranges between 0.80 and 0.92 for the subscales (51). BeSS has been validated in the normal population with the total mean 178.1 (SD 17.4) in Antonsson et al. (50) and 156.5 (SD 13.8) in Rahimifar et al. (51).

#### Quality of Life, Self-Esteem, and Psychiatric Measures

Four different self-rating procedures to assess QoL, self-esteem, and psychological symptoms.

*QoL* was assessed by *quality of life Index (QLI*) (52). The index consists of two parts with 34 items in each part. These items include questions on health and functioning, the psychological and spiritual domain, the social and economic domain, and family. The 6-point response scale ranges from 1 (very unsatisfied) to 6 (very satisfied). The total score ranges from 0 to 30 with higher scores indicating a better QoL.

*Self-esteem* was measured by “*As I see me” (AISM*). This instrument for adults is developed from an instrument that measures self-esteem in adolescents, “I think I am” (53). The questionnaire comprises 78 items that measure physical, psychological, and social self-esteem on five subscales: psychical index, skills, psychological well-being, relationship with family, and relationship with others. Positive scores reflect positive self-esteem, and negative scores reflect negative self-esteem.

*Psychological measures* (symptoms of anxiety, depression, and fatigue) were assessed by means of the Hospital Anxiety and Depression Scale (HADS) (54) and a visual analog scale for situational fatigue (55).

#### Subjective Cognitive Impairment

Subjective cognitive impairment was evaluated with *the Perceived Difficulties Scale* (PDQ) (56). This is a 20-item self-rating scale providing assessment in four different domains: attention and concentration (retrospective memory, prospective memory, planning, and organization). Each domain comprises five questions and responses are recorded on a five-point scale, ranging from “never” to “almost always.”

#### Epilepsy-Related Clinical Factors

Clinical data was obtained from interviews with the participants and from medical records. The study involved eight epilepsy-related clinical factors that could be expected to influence the severity of cognitive deficits and self-experienced complications of epilepsy: years of illness, the total number of seizures, total number of convulsive seizures (generalized tonic clonic seizures or focal seizures evolving to bilateral tonic clonic seizures), total number of focal seizures, previous epilepsy surgery, laterality of focal illness, and treatment with ASMs. Regarding the latter, we considered carbamazepine, lamotrigine, levetiracetam, and valproate.

### Ethics

Approval for the study was obtained from the regional ethical review board in Linkoping (2010/157-31). All participants gave their verbal and written informed consent before the study commenced.

### Statistical Analysis

Following visual inspection, the results were summarized using means and standard deviations. Differences between groups were evaluated in two steps. First, we employed one-way or mixed (or “split-plot”) Analysis of Variance (ANOVA) for each task. When the ANOVA yielded a statistically significant result, the Dunn–Šidák corrected *post-hoc* test was used to elucidate differences between groups. We also calculated effect sizes. The level of significance was set at *p* < 0.05. The Kendall rank correlation coefficient (τ) was used for correlations. To analyze demographic and clinical background we used non-parametric procedures (the Kruskal Wallis *H*-test or the Mann-Whitney *U*-test). IBM SPSS Statistics for Windows (v. 25.0, Armonk, NY: IBM Corp.) was used for all these calculations.

Next, we complemented the above-mentioned procedures with their corresponding Bayesian alternative. Bayesian methods can quantify the evidence in favor of any hypothesis, including the null hypothesis. These tests are here expressed as Bayes Factors (BF_10_; understood as “Bayes Factor for hypothesis 1 over hypothesis 0”). For instance, a BF_10_ equal to 4 can be interpreted as four times more support for the hypothesis of a difference compared to the null hypothesis. A BF_10_ equal to 0.25 can conversely be seen as lending four times more support for the null hypothesis in comparison to the rival hypothesis. A Bayes factor (BF_10_) larger than 3 is often considered as evidence of moderate strength in favor of the hypothesis and a BF_10_ larger than 10 indicate strong evidence. Conversely, a BF_10_ smaller than 0.3 indicate evidence of moderate strength in favor of the null hypothesis (here, the absence of a difference between groups) and a BF_10_ smaller than 0.1 suggests strong evidence for the null. Values between 0.3 and 3 provide anecdotal evidence. It could be noted that BF_10_ at times becomes very large. When this happens, BF_10_ is expressed as log BF_10_. We used JASP v. 0.11.1 to compute the Bayesian results (57).

## Results

The results, including test statistics, are presented in Tables 2–4 and Supplementary Tables 1–9. When ANOVAs were used, results are detailed in the text below.

**Table 2:** Demographics and clinical background data for participants in a study on young adults with epilepsy in Sweden (means, standard deviations and range).

**Measure**	**Focal epilepsy**	**Generalized epilepsy**	**Healthy controls**	**Statistics**
Age (years)	25.2 (4.01; 18–33)	26.4 (5.05; 20–35)	25.5 (4.21; 18–35)	NS
Education (years)	13.4 (2.3; 10–19)	13.2 (1.4; 12–16)	14.5 (2.2; 12–20)	NS
Sex (Female/Male)	10/7	6/5	16/12	NS
Epilepsy duration (years)	12.5 (7.9; 1–25)	12.0 (5.5; 5–20)	NA	NS

*NA, Not applicable; NS, Not significant as determined by Kruskal Wallis H (Age and Education), Mann-Whitney U (Duration, Subjective Memory Problems), or χ^2^-test (Sex)*.

**Table 3:** Results regarding additional cognitive measures (means, standard deviations, and range) in a study of young adults with epilepsy in Sweden.

**Measure**	**Focal epilepsy**	**Generalized epilepsy**	**Healthy controls**	**Bayes factor (BF_10_)**	***R^2^***	**Focal vs. Generalized^a^**
Episodic memory						
RCFT: Recall	19.0 (6.8; 3–29)	20.0 (6.4; 10.5–27)	21.9 (5.7; 11.5–33)	0.40	0.01	NA^b^
RCFT: Recognition	19.1 (4.3; 8–24)	17.4 (7.0; 2–24)	21.4 (1.6; 19–24)	3.73	0.09	0.46
RAVLT Recognition	0.76 (0.17; 0.37–0.94)	0.81 (0.19; 0.31–0.94)	0.86 (0.09; 0.62–0.94)	1.11	0.04	NA^b^
Executive						
TMT A (sec)	38.2 (13.8; 20–77)	33.1 (14.3; 18–66)	22.1 (6.8; 11–32)	325.7	0.25	0.48
TMT B (sec)	84.4(34.6; 37–164)	107.5 (70.7; 45–285)	55.3 (26.0; 28–72)	23.7	0.16	0.59
Attention						
Digit span forward	6.0 (0.9; 5.0–7.5)	5.5 (0.6; 5.5–6.5)	6.6 (1.1; 5.0–9.0)	44.2	0.18	0.66
Digit span backwards	4.8 (1.1; 3.5–6.5)	5.0 (0.9; 3.5–6.5)	5.5 (0.8; 4.5–7.5)	1.93	0.06	0.39
Listening span	3.5 (0.6; 2.2–4.5)	3.5 (0.4; 2.8–4.3)	4.0 (0.6; 3.25–5.5)	11.74	0.14	0.36
Visuospatial						
RCFT: Copy	34.5 (1.6; 32.0–36.0)	33.7 (2.4; 29.5–36.0)	34.8 (1.8; 27.5–36.0)	0.39	0.01	NA^b^
WAIS-IV block design	43.1 (12.7; 21–64)	43.3 (12.7; 18–56)	52.5 (10.6; 30–66)	4.2	0.09	0.35
Language						
WAIS-IV vocabulary	33.6 (10.1; 16–56)	32.6 (9.1; 17–49)	41.7 (7.3; 24–60)	19.2	0.16	0.36
COWAT verbal fluency	37.2 (15.7; 16–72)	34.7 (12.7; 11–57)	43.1 (11.3; 22–62)	0.7	0.02	0.39
BESS total	122.6 (39.2; 59–190)	122.6 (35.6; 66–181)	162.0 (21.0; 117–193)	486.8	0.65	0.16

*R^2^ denotes model averaged variance explained.*

a *Denotes Bayesian post-hoc test.*

b *Not applicable as there was no statistically significant Group main effect in univariate or Bayesian ANOVA.*

*RCFT, Rey Complex Figure Test; RAVLT, Ray Auditory Verbal Learning Test; TMT, Trail Making Test; WAIS-IV, Wechsler Adult Intelligence Scale, Fourth Edition; COWAT, Controlled Oral Word Association Test; BESS, Assessment of Subtle Language Impairments*.

**Table 4:** Results regarding quality of life, self-esteem, and psychiatric measures in a study of young adults with epilepsy in Sweden (means, standard deviations and range).

**Measure**	**Focal epilepsy**	**Generalized epilepsy**	**Healthy controls**	**Bayes factor (BF_10_)**	***R^2^***	**Focal vs. Generalized^a^**
QLI	19.3 (3.0; 9.5–23.6)	22.7 (2.0; 20.7–26.2)	22.2 (3.6; 10.6–26.8)	41.52	0.23	7.20
AISM	55.9 (31.4; 53–106)	89.8 (21.1; 58–118)	100.9 (19.4; 52–133)	1672.4	0.42	3.97
HADS anxiety	7.2 (3.4; 4–13)	6.2 (4.8; 0–13)	3.9 (2.6; 0–12)	9.98	0.13	0.60
HADS depression	2.5 (0.9; 2–8)	2.1 (1.4; 0–4)	2.2 (1.6; 0–6)	1.17	0.04	1.78
Fatigue	54.7 (25.2; 9–80)	58.3 (27.5; 12–100)	52.4 (23.6; 0–80)	0.39	0.01	0.45

*R^2^ denotes model averaged variance explained.*

a *Denotes Bayesian post-hoc test.*

*QLI, Quality of Life; AISM, self-esteem; HADS, Hospital Anxiety and Depression Scale*.

### Demographics and Clinical Characteristics

A total number of 28 participants with epilepsy and 28 healthy controls matched for gender, age, and education level were included and completed the neuropsychological examination and the questionnaires. The demographic data is summarized in Table 2. The results for QoL were acquired from 26 participants and 20 healthy controls. Information regarding self-esteem was acquired from 26 participants and 24 healthy controls. Seven participants with focal onset seizures but none of the participants with generalized seizures had been treated with brain surgery. All epilepsy participants used ASMs, many of them in combination therapy. The most common drug was lamotrigine followed by levetiracetam, valproate, topiramate, and carbamazepine. Seven participants were treated by monotherapy, 16 by ditherapy, and 5 participants were treated with 3 different substances.

### Results Concerning Cognitive Performance

Participants with epilepsy performed worse than controls within all cognitive domains, with no significant differences noted between patient subgroups or laterality of focal epilepsy. Detailed results below.

#### Episodic LTM

**Figure 1:**
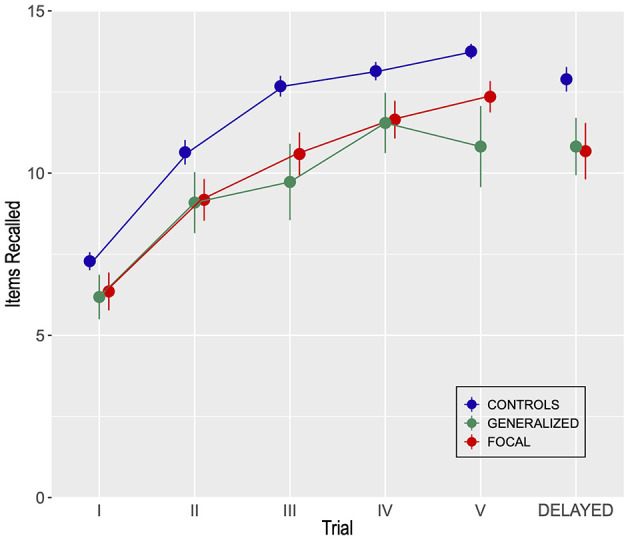
Performance for two groups of participants with generalized (*n* = 11) or focal (*n* = 17) epilepsy and for a control group (*n* = 28) and on the Rey Auditory Verbal Learning Test (RAVLT). Error bars indicate SEM.

Verbal episodic long-term memory was impaired in participants with both generalized and focal epilepsy compared to the healthy control group as assessed by number of items recalled (of 15 possible) on the Rey Auditory Verbal Learning Test (Figure 1). A Bayesian repeated measures ANOVA corroborated these results and indicated that the model comprising the main effects (i.e., Group and Trial), explaining 79% of the variability, offered the most plausible representation of the data (Supplementary Table 1). There were no significant differences between focal and generalized epilepsy. A Bayesian *post-hoc* test for the difference between focal and generalized epilepsy yielded a BF_10_ = 0.23, hence supporting the absence of differences between epilepsy groups in terms of RAVLT.

A mixed Group by Trial ANOVA comprising learning trials one to five and delayed recall indicated statistically significant effects of Group: *F*_(2, 53)_ = 5.53, MSE = 24.5, *p* < 0.01, η^2^ = 0.17. Also, the effect of Trial was significant: *F*_(5, 265)_ = 118.5, MSE = 1.88, *p* < 0.001, η^2^ = 0.69. Finally, the Group by Trial interaction was marginally significant: *F*_(10, 265)_ = 1.73, MSE = 1.88, *p* = 0.07, η^2^ = 0.06. The figure shows the number of words recalled across five study-recall trials and a trial involving delayed recall.

Results from the recognition part of RAVLT are summarized in Table 3 (means and Bayesian results) and Supplementary Table 2 (ANOVA). The results failed to disclose credible or statistically significant differences between subgroups with epilepsy. The Copy and Recall conditions of *RCFT* did not differ between groups. Controls recognized more graphic elements than the epilepsy groups, but the comparison between focal and generalized epilepsy gave inconclusive support for the null hypothesis of no difference (BF_10_ ≈ 0.4).

#### Executive Functions

Participants with epilepsy exhibited impairment of executive functions (Table 3; Supplementary Table 3), most obvious in generalized epilepsy using significantly longer time for TMT B, compared to controls. Although the Bayesian *post-hoc* tests of the difference were in favor of the absence of differences between epilepsy subtypes (BF_10_ <1), the result remained inconclusive.

#### Attention

**Figure 2:**
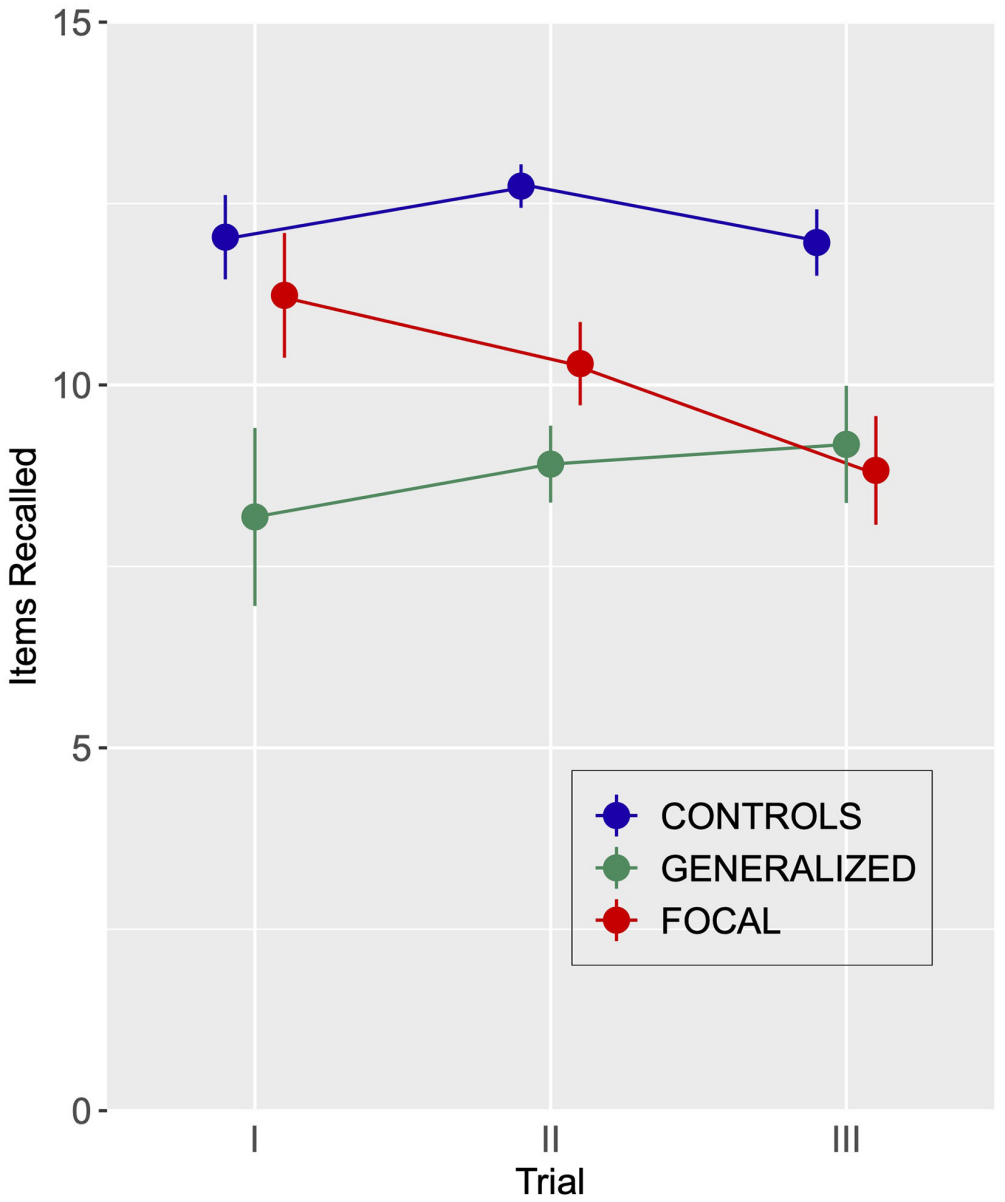
Performance for participants with generalized (*n* = 11) or focal (*n* = 17) epilepsy and healthy controls (*n* = 28) on the Auditory Consonant Trigram Test. The number of trigrams recalled following delays of 9, 18, and 36 s are shown. Error bars indicate SEM.

Attention was negatively affected in both generalized and focal epilepsy study participants compared to controls on the *Auditory Consonant Trigram, Digit Span Forward*, and *Backward and the Listening Span*, with no differences between epilepsy subtypes (Figure 2, Table 3; Supplementary Tables 4, 5).

The univariate mixed repeated measures ANOVA, Group yielded a significant effect: *F*_(2, 52)_ = 17.04, MSE = 9.7, *p* < 0.001, η^2^ = 0.40. The Trial effect was non-significant (*F* < 1.0). The same goes for the Group by Trial interaction: *F*_(2, 52)_ = 1.90, MSE = 6.3, *p* = 0.12, η^2^ = 0.07.

#### Visuospatial Functions

Participants with epilepsy had lower results on the block design task compared to healthy controls with no difference between focal and generalized epilepsy (Table 3; Supplementary Table 6). All three groups performed on a pair on RCFT copy.

#### Language

**Figure 3:**
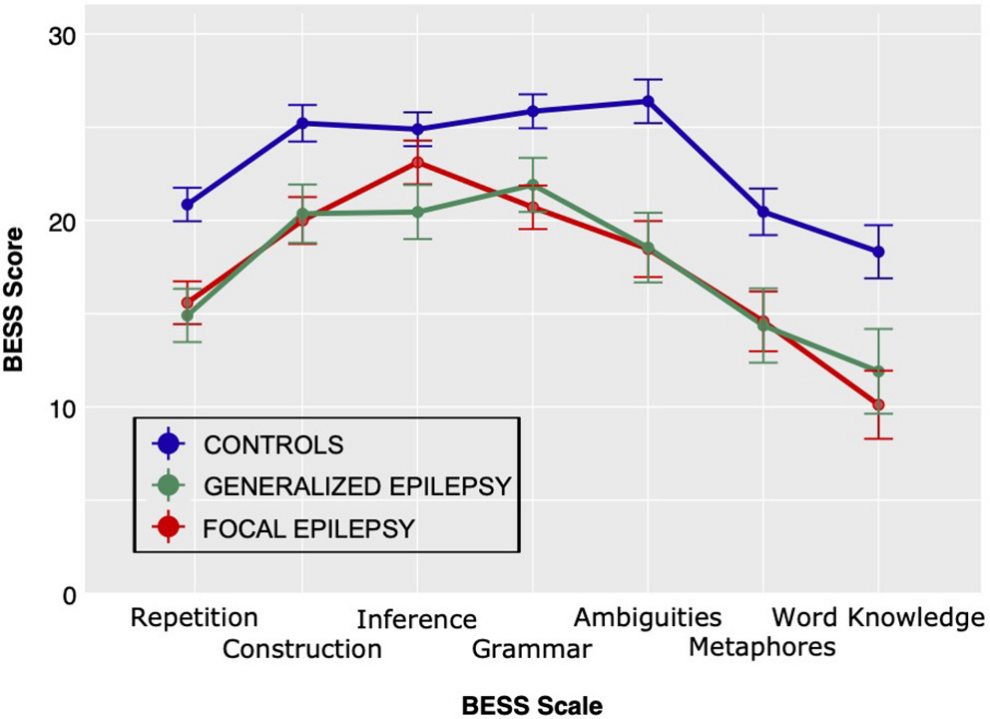
Performance for participants with generalized (*n* = 11) or focal (*n* = 17) epilepsy and healthy controls (*n* = 28) on the BeSS assessment (Assessment of subtle language impairments).

Participants with epilepsy scored lower than healthy controls on the different language tests (Table 3 and Figure 3; Supplementary Table 7), but there were no significant differences between the epilepsy subtypes. A mixed Group by BESS Scale ANOVA produced a statistically significant main effect of Group: *F*_(2, 53)_ = 11.74, *MSE* = 132.2, *p* < 0.001, η^2^ = 0.31. Similarly, the effect of *BeS*S Scale was significant: *F*_(6, 318)_ = 38.7, *MSE* = 17.05, p <0.001, η^2^ = 0.42. However, the Group by *BeSS* Scale interaction did not unequivocally evidence statistical significance: *F*_(12, 318)_ = 1.62, *MSE* = 17.05, *p* = 0.08, η^2^ = 0.06.

The Bayesian ANOVA confirmed these results and *post-hoc* tests favored the null hypothesis of no difference between epilepsy groups (Supplementary Table 9). Patients with focal epilepsy starting in the left hemisphere scored similar to the other epilepsy patients (all *t*'s <1.04, and values of BF_10_ 0.43–0.61).

#### Quality of Life, Self-Esteem, and Psychological Measures

Study participants with focal epilepsy had significantly decreased QoL compared to participants with generalized epilepsy. For the latter, QoL was similar to healthy controls. Self-esteem was lower in both epilepsy groups. Furthermore, participants with focal epilepsy scored significantly lower on self-esteem than participants with generalized epilepsy (Table 4; Supplementary Table 9). Both epilepsy subtypes reported slightly increased levels of anxiety. Ratings of depression and fatigue were equal to healthy controls. However, it should be noted that none of the study participants reached the recommended threshold for clinically significant problems (the HADS anxiety scale > 15).

We also examined correlations between the above-mentioned measures, cognition, and disease-related factors. The correlation between cognition and QoL and self-esteem were varying (τ varying between −0.17 and 0.35) and there was one statistically significant exception: TMT A and QoL (τ = −0.27, *p* = 0.01, BF_10_ = 5.8). In contrast, quality of life correlated with HADS Anxiety (τ = −0.25, *p* = 0.02, BF_10_ = 3.4) and HADS Depression (τ = −0.39, *p* < 0.001, BF_10_ = 141.0). QoL also correlated with number of focal seizures (τ = −0.43, *p* = 0.005, BF_10_ = 18.5). Self-esteem similarly was correlated with the total number of convulsive and focal seizures (τ = −0.43, *p* = 0.002, BF_10_ = 8.8).

Hence, psychomotor speed, depression, and the severity of illness all seem to contribute to quality of life. In an exploratory manner, we corroborated this impression by running a hierarchical linear multiple regression model. Number of seizures was entered in a first step and TMT A, HADS and depression were entered in a second step. This model explained 53% of the variation in QoL, retaining all three predictors.

### Subjective Cognitive Difficulties

**Figure 4:**
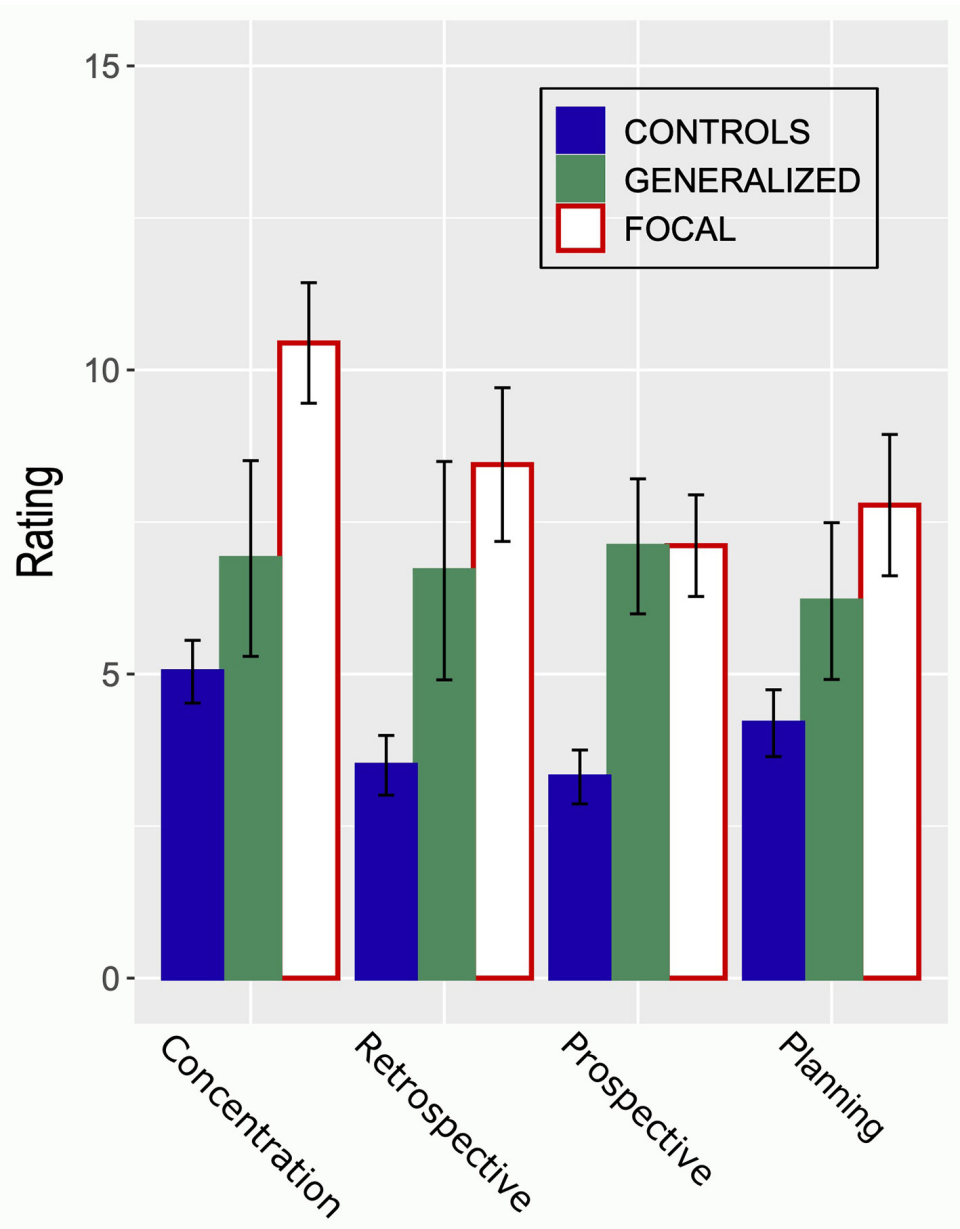
The figure shows ratings provided by a control group (*n* = 28) and two groups of participants with generalized (*n* = 11) or focal (*n* = 17) epilepsy on the four scales of the Perceived Difficulties Questionnaire (PDQ). A higher rating indicates more self-perceived problems with respect to concentration, retrospective memory, prospective memory, or planning. Error bars indicate SEM.

Participants with epilepsy reported more cognitive difficulties than healthy controls (PDQ) in all four domains: concentration, retrograde memory, prospective memory, and planning and organization (Figure 4). Except for a correlation between the PDQ total score and HADS Anxiety (τ = 0.29, *p* = 0.004; BF_10_ = 15.38), there was no association between PDQ and the other variables included in this study.

Self-perceived cognitive difficulties, as measured with the *Perceived difficulties questionnaire*, differed among domains between participants with generalized (*n* = 11) or focal (*n* = 17) epilepsy and healthy controls (*n* = 28) as evidenced by a main effect of Group [*F*_(2, 50)_ = 9.71, MSE = 44.3, *p* < 0.001] and Domain [*F*_(3, 150)_ = 5.94, MSE = 4.2, *p* < 0.001] in a mixed ANOVA. The Group by Domain interaction effect received circumstantial support: *F*_(6, 150)_ = 1.91, MSE = 4.2, *p* = 0.082. This outcome was confirmed by a Bayesian ANOVA, a model incorporating the effects of Group and Domains outperformed models involving Group or Domain, or a model incorporating the Group by Domain interaction: BF_10_ = 129179.6; *r*^2^ = 0.73. *Post-hoc* tests (both univariate and Bayesian) confirmed differences between controls and epilepsy groups; the differences between the two epilepsy groups were inconclusive (BF_10_ = 0.9). A higher rating indicates more self-perceived problems with respect to concentration, retrospective memory, prospective memory, or planning.

### Epilepsy-Related Clinical Factors

The following epilepsy-related clinical factors were included: years of illness, the total number of seizures, number of convulsive seizures, number of focal seizures, previous surgery, and laterality of epilepsy focus. The total number of convulsive seizures were related to cognitive measures of episodic memory and language. No other associations were noted.

The total number of convulsive seizures was correlated to RAVLT delayed recall (τ = −0.37, *p* = 0.02; BF_10_ = 7.8) and delayed recognition (τ = −0.35, *p* = 0.04; BF_10_ = 3.8). One participant was an outlier and hence removed from the analysis. However, including this participant did not alter the interpretation.

## Discussion

### Cognition

This study shows that patients with both generalized and focal epilepsy have lower mean results in several cognitive domains, compared to healthy matched controls. This result was consistently noted across most of the cognitive domains as episodic LMT, executive function, attention, visuospatial function, and language, although estimates of effect size were in the weak to moderate range (varying between 0.24 and 1.07). Hence, there were no clear differences between generalized and focal epilepsy in terms of cognitive functions. These results were corroborated by Bayesian analyses, which provided explicit evidence in favor of the lack of differences between generalized and focal epilepsy.

#### Memory and Attention

There is a lack of understanding about how differences and similarities between different subtypes of epilepsy impact on both cognition and psychosocial functioning. Our study demonstrates an interesting result: Similar cognitive profiles in focal and generalized epilepsy as both groups differed from controls at a similar level with respect to all examined cognitive domains. Previous studies have demonstrated deficits regarding attention and executive functions in generalized epilepsy (58). This study confirmed deficits in these domains, as well as for episodic LTM and language functions. The present results are in line with those from our previous publication on patients with generalized epilepsy in which we demonstrated impaired language abilities, connected to aberrations in the cerebral default mode network evaluated by functional magnetic resonance imaging (fMRI) (9). In generalized epilepsy we found an imbalance in the form of inadequate suppression of the default mode network during a sentence reading task.

The ILAE classification from 2017 regards epilepsy as a network disease; focal seizures start in a lateralized network while generalized seizures start in a more widespread network involving both hemispheres (59). Cognition is considered to be dependent on complex networks in the brain, connecting the different cortical and subcortical modules through axonal pathways. These pathways and connections form an infrastructure that different cognitive functions rely upon. Cognitive functions are not strictly localized in the brain but rather reflect dynamic effects of distributed networks that are dependent on cortical and subcortical connections as exemplified by results from axonal direct electrical stimulation performed during neurosurgery giving rise to the theory of hodotopy. These large-scale distributed networks are susceptible to lesions on cortical or subcortical levels, as well as physiological alterations (60–62). The new classification mirrors that the exact localization of the epileptic focus is of less importance for the consequences of the disease than the networks affected. The life-history of the individual epilepsy patient and the variability of consequences of the seizure disorder may also influence the cognitive profile of the individual, in addition to localization and epilepsy diagnosis (63). For adults with temporal lobe epilepsy, three different cognitive phenotypes have been described, i.e., intact cognition, global impairment affecting all examined cognitive functions or focal impairment, most often affecting language, memory or executive function (64, 65). In this recent important work the classical lesion-based profile was found only in 30.1% of the adults with temporal lobe epilepsy, while a higher proportion exhibited a cognitive profile comparable with healthy controls (64). A minor proportion exhibited a generalized impaired cognition, a finding that might explain part of the similarities between the two groups in the present study. Accordingly, different forms of epilepsy may present with similar cognitive problems.

A first implication of this new model of explaining cognitive impairment in epilepsy and the overall similarity between epilepsy subtypes regards the clinical management of epilepsy in young adults. The participants had no clinical diagnosis or even recognition of cognitive deficits before entering the study, but many revealed a subtle dysfunction of cognition which was not recognized in the clinic. Hence, clinicians should be aware of the presence of mild to moderate, often non-overt, cognitive problems in epilepsy patients, regardless of the specific epilepsy subtype. Whilst these cognitive deficits, whenever they appear, most often are not grossly incapacitating, they can be expected to interfere with academic performance, work efficacy, and leisure activities. Also, there are no simple rules of thumb that can be applied to a specific epilepsy type. In comparison to differences between epilepsy groups, the constellation of cognitive problems varies considerably more between individuals and needs to be addressed at the individual level for personal health care.

Further support for the role of network functioning is that memory impairments have been shown regardless of the presence of a lesion detected with MRI, supporting the notion that the neuropsychological impairments depend less on the structural lesion and more on the underlying network disease (66). There are now considerable evidence that challenge the classical lesion model of understanding cognition in focal epilepsy (31), which is in line with the result of the present study. Hermann et al. suggest in their review an expanded framework for assessment of cognitive risks in epilepsy, not only including etiology, comorbidities, and treatment, but also genomic, medical, social, and life-style factors should be addressed for a more comprehensive evaluation (31).

Previous research has shown that attention networks are affected in epilepsy (67). Interictal epileptiform discharges impair performance of attention tasks, such as divided attention tasks (68). Interictal discharges negatively affect psychomotor speed, attention abilities in mathematic but not language among children with idiopathic epilepsy (69). In addition to episodic LTM and language functions, it is therefore not surprising that individuals with epilepsy evidenced obvious difficulties with tasks addressing attention, and short-term memory, including working memory. Indeed, the single task that produced largest differences between epilepsy subtypes and controls was the *Auditory Consonant Trigrams* task. This procedure highlights *forgetting* in working memory and along with the deficits seen in the listening span task, which focuses on active processing in working memory, these results indicate that problems related to attention and working memory need more careful consideration in future neuropsychological and clinical studies.

It could tentatively be suggested that some recent developments in memory research cast light upon the question why attention and working memory are affected in epilepsy. Cowan proposed that also working memory uses representations in LTM (70). Working memory is seen as temporal activation of a small part of LTM. Hence, at the level of underlying representations, long-term and working memory share many similar neural processes but when it comes to actual, task-driven processing, they employ many different processes. This model not only works well with many recent demonstrations of neural similarities between perception, working memory, and LTM (71), but also could be expanded to account for cognitive similarities, rather than differences, between different clinical presentations of epilepsy.

Memory representations are considered as bundles of features (72). When long-term representations are deranged, the memory traces contain less features. Hence, working memory is negatively affected or at least requires more attentional resources. Previous results from our group (73–75) as well as other (76) show that increased demands for intellectual performance in difficult tasks engage neural networks, according to a neural efficiency hypothesis (77). As a result, cognitive activities that differently depend on representations in LTM are negatively affected. In epilepsy, this would include episodic memory, but also semantic memory, and activities involving the use of complex language (e.g., BeSS) and working memory (e.g., Listening span, the Auditory Consonant Trigrams Task). The putative damage to long-term representations in epilepsy may occur in at least two ways. As briefly hinted above, white matter-based communication between neural modules would suffer due to seizures and sub-clinical epileptic discharges. As a result, features would be fewer and less integrated. Thus, the damage to representations would be the direct consequence of epilepsy being a disorder of brain communicative networks. However, derangement of representations could also be an effect of damage to a specific process. A fundamental aspect of many cognitive activities as perception, working memory and LTM, is the ability to discern subtle changes in the environment, often denoted as pattern separation (78). Pattern separation ascertains that similar cortical representations are updated to account for important changes in the environment. If pattern separation suffers, representations in LTM would lose in fidelity and many cognitive processes would suffer (71). It is obvious how such a deficit would interfere with performance in focal temporal lobe epilepsy, since pattern separation is dependent upon the medial temporal lobe (79). However, the medial temporal lobe receives input from and sends back information to many areas of the brain, including the visual and auditory temporal lobe association cortical areas, the prefrontal cortex, the parietal cortex, the cingulate, and the amygdala. Hence, “non-focal” seizure disorders, such as generalized epilepsy that interferes with network communication could produce similar effects.

Self-reported memory problems did not correlate to actual cognitive deficits which is in accordance with previous research. Self-reported memory and other cognitive problems are weakly related but self-reports correlate with measures of mood and emotional distress (2). Since the latter are amenable to conventional treatment of mood and anxiety disorders, clinicians must be aware of the fact that self-reports of cognitive failures often are proxies for psychological distress.

#### Language

An interesting finding was the abundance of subtle problems regarding language and semantic memory in the two epilepsy subtypes. Language related cortical regions are connected through white matter tracts and these also connect to regions of higher cognitive functions, creating a complex and sensitive network that can be affected by seizures or epilepsy related factors such as ASMs. In a literature review by Bartha-Doering and Trinka in studies of adults with epilepsy the results varied from intact language function to impairments in several domains of language (17). The studies were heterogeneous with different populations and used different methods. Dutta et al. (79) observed that there are voids regarding research on language in epilepsy other than temporal lobe epilepsy. This group also emphasized that most studies on language in epilepsy used aphasia tests which may not be appropriate for this group as more complex language functions should be examined in epilepsy (79). Indeed, BeSS demonstrated differences between both epilepsy groups and the control group on several language functions (BeSS 1, 2, 5, 6). These tests include sentence repetition, sentence construction, understanding ambiguities and comprehension of metaphors. It could be argued that an important component in all these sub-tests are general aspects of language comprehension and short-term memory. These associated functions seem to be equally, or almost equally affected in both subtypes of epilepsy. Apart from these similarities, we also found a few differences for specific groups, however difficult to interpret: Patients with generalized epilepsy had a statistical significantly lower result on BeSS 3 testing inference or the ability to understand and draw conclusions from a text. There was also a statistically non- significant trend for participants with focal epilepsy who had lower results for BeSS 4 and 6, testing comprehension of logistic grammatical constructions and definition of words. It appears that drawing conclusions based on subtle hints in a text was more challenging for the group with generalized epilepsy. The individual need to understand what is implied in the text and make a judgement of what is the most probable correct answer, also taking previous experience into account. This decision making certainly involves both frontal and parietal cortical areas, as well as subcortical areas related to long term memory. Be SS 4 reflects the general component of language comprehension, and BeSS 6 the lexical language component and vocabulary of an individual.

BeSS has the disadvantage of being available in Swedish and Farsi only. However, it is more demanding than most clinical aphasia test batteries and includes tasks that might be critical for individuals with mild-to-moderate language impairments (e.g., understanding grammatically complex text and drawing inferences from prose). Hence, the usefulness of BeSS demonstrated here could hopefully spur interest in translating the test to other languages.

Epilepsy may impact not only the cortical language areas but also the subcortical language network and white matter tracts. Caplan et al. demonstrated that language was affected in children with both localized epilepsy and absence epilepsy (80). Verbal fluency impairments have been shown in both left-sided and right-sided temporal lobe epilepsy (81). In the present study, language was affected for young adults with generalized as well as focal epilepsy and we could not find language differences between participants with left or right hemisphere foci. These findings again add weight to the view of epilepsy as a network disorder. Pulvermuller proposed that both cognitive and linguistic processes are modeled by activity dynamics in action perception circuits between specific regions, and that knowledge is organized in the dynamics within, and between such connections (82). The authors of that article argue that “the network models and the concept of distributionally-specific circuits, can account for some previously not well-understood facts about the cortical ‘hubs' for semantic processing and the motor system's role in language understanding and speech sound recognition.” Also, the semantic system is very large extending into several non-linguistic memory systems and cognitive functions (83). This could partly explain the similar results in individuals with epilepsy regardless of classification and foci.

#### Quality of Life

Given the limited number of participants and the exploratory nature of this model, these results should be interpreted with great caution. As described in the section Results, a model involving TMT A (a measure of motor, perceptual and cognitive speed), along with depression and the number of seizures could account for 53% of the variability in self-ratings of QoL. This is perhaps even more surprising as none of our participants showed gross alterations regarding the predictors in the model. In the context of more abundant seizure activity, even modest increase in depression and modest slowing of performance may impinge upon QoL.

Self-esteem was affected in focal epilepsy and to a lesser degree in generalized epilepsy. Looking at associations between self-esteem and other variables studied here, only the number of seizures showed covariation (explaining 24% of self-esteem scores). In both epilepsy groups QoL apparently was influenced by the current intensity of the manifest seizure disorder.

### Epilepsy-Related Clinical Factors

Interestingly, the number of seizures (all subtypes) were not predictive of cognitive performance in our material. Cognition, here, apparently is more related to the presence of epilepsy, not disease-modifying factors as seizure-frequency or use of ASMs. On the other hand, our results suggest that the frequency of convulsive seizures (in both focal and generalized epilepsy) add to impairment regarding episodic LTM. In contrast, the frequency of focal seizures, not leading to convulsive seizures, was not affecting cognitive performance. Complete remission of seizures seems to predict less cognitive impairment. It should be kept in mind, though, that the limited number of participants could obscure the additional contribution of focal seizure frequency to the manifestation of focal epilepsy. The effects of seizure frequency on cognition have been examined in several studies (84), with varying outcomes. At any rate, the association between the frequency of convulsive seizures and memory could alert the clinician to a risk factor that may allow for intervention.

### Limitations

Limitations of the study include the retrospective nature and the fact that the sample size of the present study was low and the sample was heterogeneous. The group with focal epilepsy included not only temporal lobe epilepsy but also other localizations of the focus in both hemispheres. Some participants with focal epilepsy had epilepsy surgery before the inclusion which is a confounding factor.

## Conclusion

The present study indicates that cognitive performances are negatively affected in a similar way regarding both cognitive profiles and magnitudes in patients with generalized and focal epilepsy. Our study confirms the recently established paradigm that epilepsy is a network disorder and it is therefore not possible to predict cognitive function by the epilepsy diagnosis or by the localization of the epileptogenic focus. Cognitive impairment, including subtle language dysfunction, is common among epilepsy patients and must therefore be acknowledged. In the long run, failure to detect and help patients with the impairments of cognition may impinge upon academic performance, work, and social relations.

## Data Availability Statement

The raw data supporting the conclusions of this article will be made available by the authors, without undue reservation.

## Ethics Statement

The studies involving human participants were reviewed and approved by Ethical Review Board in Linköping. The patients/participants provided their written informed consent to participate in this study.

## Author Contributions

HG, A-ML, ME, AM, and TK contributed to conception and design of the study. HG and TK organized the database, performed the statistical analysis, and wrote the first draft of the manuscript. HG, A-ML, PV, AF, AM, and TK wrote sections of the manuscript. All authors contributed to manuscript revision, read, and approved the submitted version.

## Funding

Funding was received (ALF) from the County Council of Östergötland.

## Conflict of Interest

The authors declare that the research was conducted in the absence of any commercial or financial relationships that could be construed as a potential conflict of interest.

## Publisher's Note

All claims expressed in this article are solely those of the authors and do not necessarily represent those of their affiliated organizations, or those of the publisher, the editors and the reviewers. Any product that may be evaluated in this article, or claim that may be made by its manufacturer, is not guaranteed or endorsed by the publisher.
