# Supplementary material for: Similar Profile and Magnitude of Cognitive Impairments in Focal and Generalized Epilepsy: A Pilot Study
Source: Front Neurol. 2022 Jan 12;12:746381. doi: 10.3389/fneur.2021.746381 (PMC8790571; doi:10.3389/fneur.2021.746381)
Supplement: Supplementary file 1 [file Table_1.pdf]

Supplementary material for:

Similar profile and magnitude of cognitive impairments in focal and generalized epilepsy: a pilot study

Helena Gauffin, Anne-Marie Landtblom, Patrick Vigren, Maria Engström, Andreas Frick, Anita McAllister, Thomas Karlsson

Supplementary Table S1

JASP summary output, Bayesian repeated measures ANOVA; RAVLT

Model Comparison

Models	P(M)	P(M|data)	Log BFM)	Log(BF10)	error %
Null model (incl. subject)	0.200	1.629e -72	-163.912	0.000	xx
TRIAL + EPI_GROUP	0.200	0.641	1.968	164.854	1.350
TRIAL + EPI_GROUP + TRIAL✻EPI_GROUP	0.200	0.283	0.455	164.034	2.150
TRIAL	0.200	0.076	-1.112	162.720	0.556
EPI_GROUP	0.200	1.132e -71	-161.973	1.939	0.845

Note. All models include subject

Analysis of Effects

Effects	P(incl)	P(incl|data)	Log(BF incl)
TRIAL	0.600	1.000	∞
EPI_GROUP	0.600	0.924	2.093
TRIAL✻EPI_GROUP	0.200	0.283	0.455

Supplementary Table S2

Additional episodic long-term memory measures: univariate ANOVA results

Measure	*F*	*P*	Effect Size
Rey Complex Figure			
Recall	1.3	0.28	0.05
Recognition	4.5	0.02	0.15
RAVLT Recognition	2.4*	0.12	0.10

Note. Effect size = η_p^2. *Welch correction for homogeneity violation.

Supplementary Table S3

Executive functions: univariate ANOVA results

Measure	*F*	*p*	Effect Size
Trail Making Test			
TMT A	10.09[1]	< 0.001	0.29
TMT B	6.38[1]	0.007	0.22

Note. Effect size = η_p^2. *Welch correction for homogeneity violation.

Supplementary Table S4

JASP summary output, Bayesian repeated measures ANOVA; Auditory Consonant Trigrams

Model Comparison

Models	P(M)	P(M|data)	BF M	BF 10	error %
Null model (incl. subject)	0.200	1.625e -4	6.5e -4	1.000	
EPI_GROUP	0.200	0.738	11.296	4545.016	0.942
DURATION + EPI_GROUP	0.200	0.162	0.775	998.813	1.947
DURATION + EPI_GROUP + DURATION ✻ EPI_GROUP	0.200	0.099	0.440	609.389	3.242
DURATION	0.200	3.417e -5	1.4e -4	0.210	2.412

Note. All models include subject

Analysis of Effects

Effects	P(incl)	P(incl|data)	BF incl
DURATION	0.600	0.261	0.236
EPI_GROUP	0.600	1.00	3389.413
DURATION ✻ EPI_GROUP	0.200	0.099	0.440

Model Averaged R^2

		95% Credible Interval	
	Mean	Lower	Upper
R^2	0.352	0.242	0.453

Post Hoc Comparisons - EPI_GROUP

		Prior Odds	Posterior Odds	BF 10, U	error %
1	2	0.587	3.707e +6	6.311e +6	2.813e -10
	3	0.587	496.092	844.554	7.749e -9
2	3	0.587	0.748	1.273	0.001

Note. The posterior odds have been corrected for multiple testing by fixing to 0.5 the prior probability that the null hypothesis holds across all comparisons (Westfall, Johnson, & Utts, 1997). Individual comparisons are based on the default t-test with a Cauchy (0, r =1/sqrt(2)) prior. The "U" in the Bayes factor denotes that it is uncorrected.

Supplementary Table S5

Attention: univariate ANOVA results

Measure	*F*	*p*	Effect Size
Digit Span			
Forward	9.28[1]	<0.001	0.23
Backward	3.43	0.04	0.12
Listening Span	6.02	0.004	0.18

Note. Effect size = η_p^2. [1]Welch correction for homogeneity violation.

Supplementary Table S6

Results regarding visuospatial tasks

Measure	*F*	*p*	Effect Size
Rey Complex Figure , Copy	1.4	0.26	0.05
WAIS Block Design	4.54	0.02	0.15

Note. Effect size = η_p^2.

Supplementary Table S7

Additional language assessments: univariate ANOVA results

Measure	*F*	*p*	Effect Size
WAIS Vocabulary	6.77	0.002	0.20
COWAT verbal fluency	2.09	0.13	0.07
BESS Total	11.31[1]	< 0.001	0.30

Note. Effect size = η_p^2. [1]Welch correction for homogeneity violation.

Supplementary Table S8

JASP summary output, Bayesian repeated measures ANOVA; BESS

Model Comparison

Models	P(M)	P(M|data)	BF M	BF 10	error %
Null model (incl. subject)	0.200	2.059e -37	8.237e -37	1.000	
Task + EPI_GROUP	0.200	0.716	10.063	3.475e +36	0.877
Task + EPI_GROUP + Task ✻ EPI_GROUP	0.200	0.283	1.579	1.375e +36	1.957
Task	0.200	0.001	0.005	6.654e +33	0.474
EPI_GROUP	0.200	1.535e -34	6.138e -34	745.250	35.016

Note. All models include subject

Analysis of Effects

Effects	P(incl)	P(incl|data)	BF incl
Task	0.600	1.000	1.112e +14
EPI_GROUP	0.600	0.999	485.898
Task ✻ EPI_GROUP	0.200	0.283	1.579

Model Averaged R^2

		95% Credible Interval	
	Mean	Lower	Upper
R^2	0.653	0.592	0.699

Post Hoc Comparisons - EPI_GROUP

		Prior Odds	Posterior Odds	BF 10, U	error %
1	2	0.587	2.688e +8	4.577e +8	7.409e -15
	3	0.587	5.554e +9	9.455e +9	9.173e -16
2	3	0.587	0.093	0.159	1.030e -5

Note. The posterior odds have been corrected for multiple testing by fixing to 0.5 the prior probability that the null hypothesis holds across all comparisons (Westfall, Johnson, & Utts, 1997). Individual comparisons are based on the default t-test with a Cauchy (0, r =1/sqrt(2)) prior. The "U" in the Bayes factor denotes that it is uncorrected.

Supplementary Table S9

Quality of life, self-esteem, and psychiatric measures: univariate ANOVA results

Measure	*F*	*p*	Effect Size
QLI	8.31	< 0.001	0.29
AISM	14.96	< 0.001	0.42
HADS Anxiety	5.76	0.005	0.18
HADS Depression	2.90	0.07	0.10
Fatigue	1.29	0.28	0.05

Note. Effect size = η_p^2. QLI = Quality of Life; AISM = self-esteem; HADS = Hospital Anxiety and Depression Scale.
